# Visceral Leishmaniasis in Somalia: Diagnosis and Management of a Classic Case in a Resource‐Limited Endemic Setting

**DOI:** 10.1002/ccr3.72521

**Published:** 2026-04-14

**Authors:** Abdirahman Nuur Hussein, Abdirahman Omer Ali, Ridwan Zakariye Ahmed, Sihaam Mohamed Abdirahman, Ahmed Dek Abdiwahab

**Affiliations:** ^1^ College of Health Sciences, School of Medicine and Surgery Amoud University Borama Somalia; ^2^ Medical Department Borama Regional Hospital Borama Somalia; ^3^ Director of Research and Publication Office, Somaliland Medical Association (SLM‐RPO) Borama Somaliland Somalia

**Keywords:** kala‐azar, neglected tropical disease, paromomycin, rK39 RDT, sodium stibogluconate, Somalia, Somaliland

## Abstract

Visceral Leishmaniasis (VL), a severe systemic neglected tropical disease (NTD) caused by Leishmania donovani complex protozoa, poses a significant public health threat, particularly in East Africa, where it is fatal if untreated. Somalia is known to be endemic, but the true burden and programmatic challenges are poorly documented due to long‐standing conflict, population displacement, and a fragmented, resource‐constrained health system. We report the case of a 40‐year‐old male farmer from Borama, Awdal region (Somaliland), presenting with a 20‐day history of intermittent high‐grade fever, chills, progressive weakness, anorexia, and vomiting. Physical examination revealed marked hepatosplenomegaly and pallor. Laboratory investigations showed moderate anemia (Hemoglobin 9.4 g/dL), thrombocytopenia (114,000/μL), and a highly elevated ESR (50 mm/h). Mild hyperbilirubinemia was noted, likely secondary to hemolysis. Tests for malaria, tuberculosis, and enteric fever were negative. Definitive parasitological confirmation was not feasible due to local resource limitations. A rapid diagnostic test (RDT) for VL based on rK39 antigen—Kalazar Detect (Human) Rapid Test—was positive. Ultrasound confirmed significant hepato‐splenomegaly. Based on the clinical presentation, positive rK39 RDT, and exclusion of other etiologies, a diagnosis of Visceral Leishmaniasis was established. The patient was treated according to WHO regional guidelines with Sodium Stibogluconate (SSG, 20 mg/kg/day) and Paromomycin (PM, 15 mg/kg/day) for 17 days. The patient tolerated treatment well; the fever resolved within five days. At one‐week post‐therapy follow‐up, he showed significant clinical improvement. This case highlights the classic presentation of VL in a fragile setting and underscores the critical role of RDTs where parasitological confirmation is unavailable. It demonstrates the feasibility of the WHO‐recommended SSG+PM combination therapy despite systemic challenges.

## Key Clinical Message

Visceral Leishmaniasis should be suspected in patients with prolonged fever and hepatosplenomegaly in endemic areas. rK39 rapid diagnostic tests are vital where parasitology is unavailable. Standard WHO‐recommended Sodium Stibogluconate and Paromomycin therapy remains effective and feasible despite significant health system challenges in resource‐limited settings.

## 1 Introduction

Visceral leishmaniasis (VL), commonly known as kala‐azar, is a life‐threatening systemic disease caused by protozoan parasites of the Leishmania donovani complex [1]. Transmitted by infected female phlebotomine sandflies, VL is a major neglected tropical disease (NTD). The Horn of Africa bears the world's second‐highest VL burden [2]. Endemic foci are well‐documented in Sudan, South Sudan, Ethiopia, Kenya, and Uganda [3].

Somalia is recognized as endemic, particularly in southern regions, but its true public health impact remains unquantified [2]. Decades of conflict and a weakened public health infrastructure have created substantial barriers to surveillance and the management of both communicable and non‐communicable diseases in the region (3). In resource‐limited settings such as Somalia, diagnosis frequently relies on recognizing the clinical syndrome, combined with rapid diagnostic tests (RDTs), as definitive parasitological confirmation is often unavailable [1]. This case report details the management of a VL case at Borama Regional Hospital in the Awdal region, Somaliland, a self‐declared state internationally recognized as part of Somalia, illustrating the application of WHO standard protocols in a challenging environment.

## 2 Case History/Examination

A 40‐year‐old male farmer residing in the Borama district presented with a 20‐day history of intermittent high‐grade fever (up to 39°C) and chills. He reported progressive weakness, significant anorexia for 14 days, and non‐bilious vomiting.

On clinical examination, the patient appeared ill and pale (Temperature 39°C, HR 110 bpm, BP 110/70 mmHg). There was moderate conjunctival pallor but no jaundice or lymphadenopathy. Abdominal examination revealed marked splenomegaly (10 cm below the left costal margin) and hepatomegaly (5 cm below the right costal margin). Regarding nutritional status, while a formal Body Mass Index (BMI) calculation was not recorded at admission, the patient did not exhibit signs of severe wasting or cachexia. Serum albumin levels were preserved (47 g/L), suggesting that despite reported anorexia, severe malnutrition had not yet been established.

## 3 Differential Diagnosis, Investigations, and Treatment

Initial laboratory investigations (Table 1) revealed normocytic, normochromic anemia (Hb: 9.4 g/dL), thrombocytopenia (Platelets: 114,000/μL), and elevated ESR (50 mm/h). Liver enzymes (ALT/AST) were normal. However, Total Bilirubin was mildly elevated (1.7 mg/dL) with a normal Direct Bilirubin (0.6 mg/dL). In the context of splenomegaly and anemia, this indirect hyperbilirubinemia is clinically suggestive of mild hemolysis (hypersplenism) or infection‐related red cell destruction rather than hepatocellular injury.

**TABLE 1:** Baseline laboratory findings.

Parameter	Result	Reference range	Unit
WBC	5.5	4.0–11.0	× 10^9^/L
Hemoglobin	9.4	13.0–17.0	g/dL
Platelet Count	114	150–450	× 10^9^/L
ESR	50	< 20	mm/h
Random Blood Glucose (RBS)	155	75–180	mg/dL
Creatinine	0.9	Up to 1.1	mg/dL
Total Bilirubin (T.Bilirubin)	1.7	Up to 1.0	mg/dL
Direct Bilirubin (D.Bilirubin)	0.6	Up to 0.3	mg/dL
ALT (SGPT/ALT)	34	Up to 38	U/L
AST (SGOT/AST)	36	Up to 38	U/L
Alkaline Phosphatase	280	40–306	U/L
Albumin	47	38–51	g/dL
Total Protein (T. PROTEIN)	6.5	6.0–8.0	mg/dL
H.Pylori Ab	Negative	Negative	
Malaria RDT/Smear	Negative	Negative	
Widal Test	Negative	Negative	
Tuberculin Skin Test	Negative	Negative	

**FIGURE 1:**
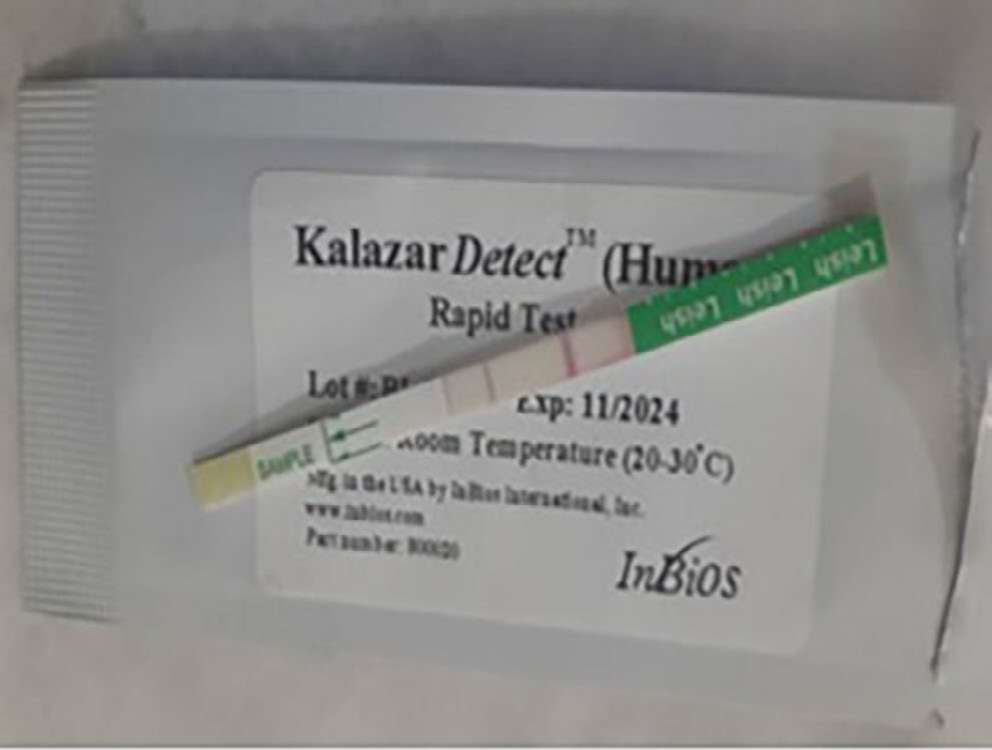
Positive result on a rapid diagnostic test (RDT) for Visceral Leishmaniasis using the rK39 antigen.

**FIGURE 2:**
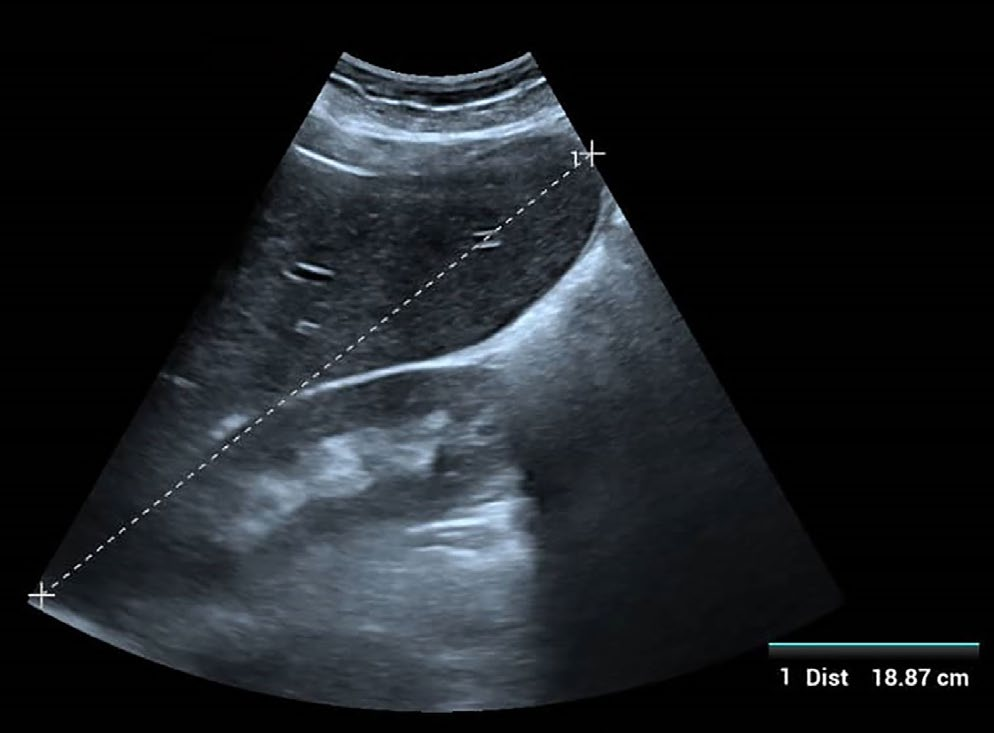
Abdominal ultrasound demonstrating hepatomegaly (liver span 18.87 cm) with homogeneous echotexture and smooth surface. No focal mass identified.

**FIGURE 3:**
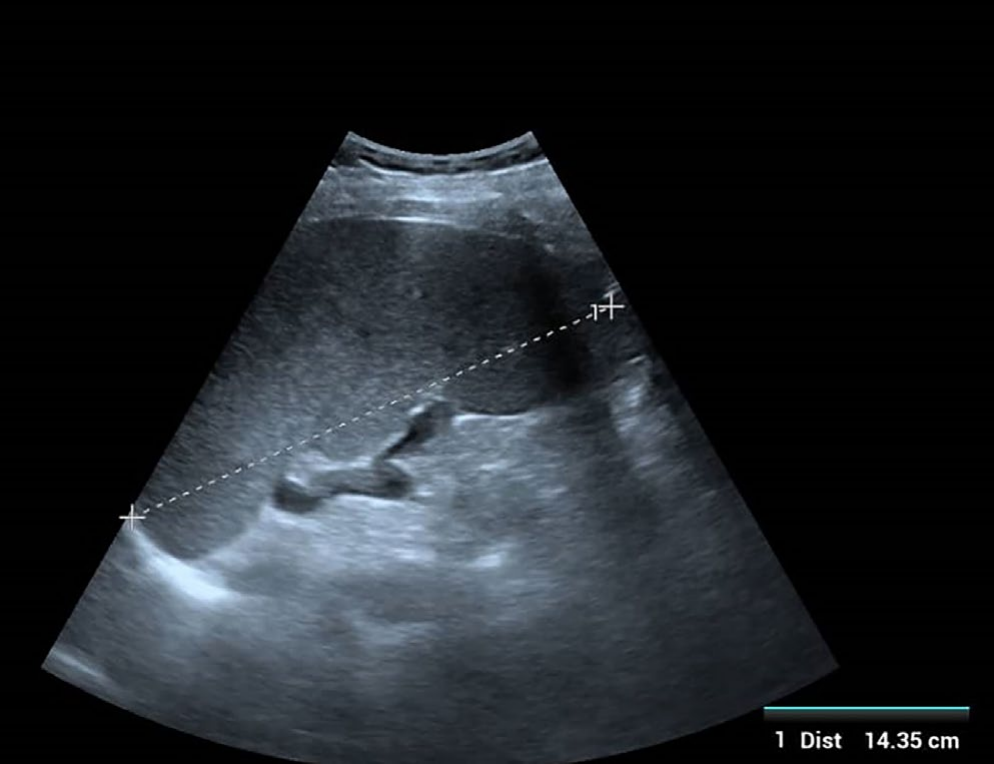
Abdominal ultrasound demonstrating marked splenomegaly (spleen length 14.35 cm) with homogeneous echotexture. No focal mass identified.

To explore the differential diagnosis of prolonged fever and hepatosplenomegaly in this endemic region, tests for malaria (RDT/Smear) were conducted, as malaria remains a significant threat in the region [4]. Additionally, tuberculosis was considered, as it often presents with extrapulmonary involvement in Borama [5]. Tests for malaria, tuberculosis (Mantoux), enteric fever (Widal), and *H. pylori* were negative. Due to the lack of specialized personnel and equipment for splenic/bone marrow aspiration, definitive parasitological microscopy was not performed. An rK39 antigen‐based rapid diagnostic test (Kalazar Detect) was positive (Figure 1). Abdominal ultrasonography confirmed hepatomegaly (liver span 18.87 cm; Figure 2) and marked splenomegaly (spleen length 14.35 cm; Figure 3) with a homogeneous echotexture.

Based on the clinical triad, origin, and positive rK39 RDT, a diagnosis of VL was established. The patient was treated with the WHO‐recommended regimen for East Africa: intravenous Sodium Stibogluconate (SSG) at 20 mg/kg/day and intramuscular Paromomycin (PM) at 15 mg/kg/day for 17 days [3, 6].

## 4 Conclusion and Results (Outcome and Follow‐Up)

Fever resolved by day five. The patient completed the 17‐day course with only mild injection site pain. At 1‐week follow‐up, he was afebrile with improved appetite. Long‐term follow‐up (6 months) was advised to monitor for relapse or post‐Kala‐azar Dermal Leishmaniasis (PKDL) per WHO guidelines (8), though the patient lives in a remote rural setting, which makes consistent surveillance challenging.

In conclusion, this case documents the successful management of classic VL in Somalia. It underscores the importance of maintaining a high clinical suspicion in patients presenting with prolonged fever and hepatosplenomegaly. Rapid diagnostic tests (rK39) proved indispensable for facilitating timely diagnosis where gold‐standard methods were unavailable. The standard WHO‐recommended SSG+PM combination therapy resulted in a favorable clinical outcome, demonstrating its feasibility even in resource‐constrained environments.

## 5 Discussion

VL remains a major NTD challenge, inflicting substantial morbidity and mortality, particularly in East Africa [1, 3]. This case exemplifies the classic clinical presentation driven by the parasite's affinity for the reticuloendothelial system [4, 7, 8]. Diagnosing VL presents significant hurdles in resource‐limited settings like Somalia, where diagnostic capacity is often minimal and other febrile illnesses are highly prevalent [1]. Furthermore, systemic deficiencies often lead to delays in identifying rare or complex conditions in this context [5, 9].

While the gold standard remains the microscopic demonstration of amastigotes [1, 7, 10], this was not feasible. In such contexts, WHO recommends a syndromic approach combined with rK39 RDTs [1, 3]. It is important to note that while rK39 RDTs demonstrate high sensitivity (> 90%) in the Indian subcontinent, studies in East Africa have shown variable sensitivity (ranging from 75% to 85%), although specificity remains excellent (1, 4). A limitation of serology is antibody persistence, which prevents its use for diagnosing relapse (1).

While newer treatments like Liposomal Amphotericin B are safer, their cost and cold‐chain requirements limit access in Somalia [3, 6, 9]. Effective management also requires post‐treatment surveillance; WHO guidelines recommend follow‐up at 6–12 months to detect potential relapse or the development of PKDL [6, 9, 11], a protocol we emphasized to the patient.

## Author Contributions

**Abdirahman Nuur Hussein:** conceptualization, methodology, project administration, supervision. **Abdirahman Omer Ali:** conceptualization, project administration, resources, supervision, writing – original draft, writing – review and editing. **Ridwan Zakariye Ahmed:** data curation, formal analysis, investigation, validation. **Sihaam Mohamed Abdirahman:** funding acquisition, methodology, validation. **Ahmed Dek Abdiwahab:** formal analysis, funding acquisition, methodology, resources, software.

## Funding

The authors have nothing to report.

## Ethics Statement

Ethical approval was secured from the appropriate Institutional Review Board (IRB) of Borama Regional Hospital. The study also received approval from the Ministry of Health and Borama Regional Hospital in the Awdal Region, Somaliland (Approval No: BRH‐261/2024). Written informed consent was obtained from the patient for the collection of clinical data and for the publication of this case report and any accompanying de‐identified images and clinical information. Patient confidentiality has been maintained throughout the preparation of this manuscript.

## Consent

Written informed consent was obtained from the patient.

## Conflicts of Interest

The authors declare no conflicts of interest.

## Data Availability

All relevant data generated or analyzed during this study are included in this published article. Further details are available from the corresponding author upon reasonable request.
